# Spatial control of robust transgene expression in mouse artery endothelium under ultrasound guidance

**DOI:** 10.1038/s41392-022-01031-w

**Published:** 2022-07-18

**Authors:** Renfa Liu, Shuai Qu, Yunxue Xu, Hanjoong Jo, Zhifei Dai

**Affiliations:** 1 Department of Biomedical Engineering, College of Future Technology, National Biomedical Imaging Center, Peking University, Beijing, China; 2 Wallace H. Coulter Department of Biomedical Engineering, Georgia Institute of Technology and Emory University, Atlanta, USA

**Keywords:** Cardiovascular diseases, Gene therapy

**Dear Editor**,

Dysfunctional vascular endothelial cells (ECs) contribute to the pathophysiology of several cardiovascular diseases, such as atherosclerosis and its life-threatening complications.^1^ Gene therapy can be a valuable approach to modulate endothelial cell function for the prevention of atherosclerosis. However, there is still a lack of method for transgene expression in vascular endothelium.^2^ Herein, an effective and specific strategy was established for noninvasive spatial control of transgene expression into the target region of the mouse artery without systemic spillover using an ultrasound and microbubble (MB) guided adeno-associated viral vector (UMGAAV) (Supplementary Fig. S1). To the best of our knowledge, this is the first report on noninvasive spatial control of transgene expression in arterial endothelium in vivo.

To establish the UMGAAV, we performed the ultrasound treatment on the mouse's left common carotid artery (LCA) while leaving the right common carotid artery (RCA) untreated as a control. To achieve the transgene expression in the mouse carotid artery, we performed ultrasound treatment using an ultrasound system equipped with a linear-array probe operating at 4.4 MHz, which is also used in clinics for carotid ultrasound imaging. Under color Doppler mode, the mouse carotid artery can be clearly visualized and the whole carotid artery can be zoomed on the image so that ultrasound can be focused on this area (Fig. 1a). After injecting MBs, the MBs can be destroyed by the ultrasound wave generated by the imaging probe, as evidenced by the “blooming” artifact in color Doppler imaging.

**Fig. 1:**
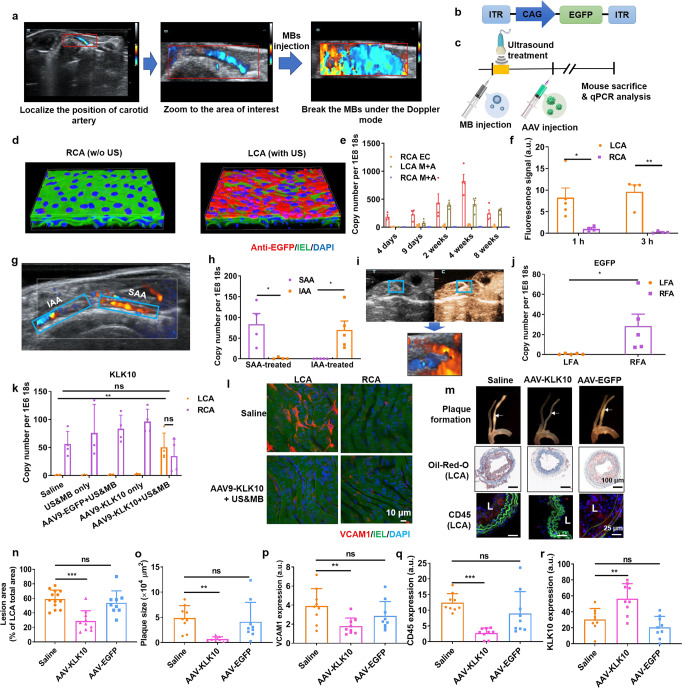
Spatial control of transgene expression in mouse artery with ultrasound. **a** The mouse carotid artery can be localized by color Doppler ultrasound imaging. By changing the size and position of color box, the ultrasound image can be zoomed to the area of interest so that ultrasound was focused to this area. After MBs injection, the MBs can be destroyed, as evidenced by the color “blooming” phenomenon. **b** AAV gene vector carrying the expression cassette for EGFP. ITR, internal terminal repeats; CAG, a hybrid construct consisting of the cytomegalovirus (CMV) enhancer fused to the chicken beta-actin promoter. **c** Illustration of UMGAAV protocol. After MB injection, ultrasound irradiation was applied to the area of interest for 30 s. Following AAV injection, the mouse was sacrificed at specific time points for qPCR analysis using 18S as an internal control. **d** Representative 3-D view of confocal assessment of EGFP expression in LCA and RCA. EGFP was immunostained with EGFP antibody (red). The DAPI staining marks cell nuclei (blue). The green autofluorescence was from the internal elastic lamina (IEL). **e** Transgene expression of EGFP in endothelial cells (ECs) and medial and adventitial layer (M + A) of LCA and RCA at different time points post AAV injection. The time interval between MB and AAV injection was ~5 min. **f** Quantification of AAV fluorescence in LCA and RCA. Fluorescently-labeled AAV was injected after ultrasound treatment in LCA and the mice were sacrificed at 1 h and 3 h post injection. The AAV fluorescence in the arteries were quantified. **g** Representative image of mouse suprarenal abdominal aorta (SAA) and infrarenal abdominal aorta (IAA) with color Doppler imaging. **h** Transgene expression of EGFP in SAA and IAA when SAA or IAA was treated with ultrasound. **i** The mouse femoral artery can be detected with contrast-enhanced ultrasound imaging. After focusing the ultrasound to the major branch of femoral artery, the MBs can be selectively destroyed with color Doppler ultrasound imaging. **j** Transgene expression of EGFP in mouse femoral artery when right femoral artery (RFA) was treated while the left femoral artery (LFA) was untreated. **k** Expression of KLK10 in arterial endothelium in mice with different treatments. Saline, mice were injected with saline only; US&MB only, LCA were treated with ultrasound after MB injection; AAV9-EGFP + US&MB, the mice were injected with AAV9 expressing EGFP after ultrasound treatment; AAV9-KLK10 only, the mice were injected with AAV9-KLK10 without ultrasound treatment; AAV9-KLK10 + US&MB, the mice were injected with AAV9-KLK10 after ultrasound treatment. **l** *En face* VCAM1 staining of carotid artery. The arteries were immunostained with VCAM1 antibody (red). The DAPI staining marks cell nuclei (blue). The green autofluorescence was from IEL. **m** Representative bright-field images of aortic trees (top lane) and the Oil-Red-O staining (middle lane) and anti-CD45 immunostaining (bottom lane) of frozen sections prepared from the middle parts of these arteries. CD45 was immunostained in red. The DAPI staining marks cell nuclei (blue). The green autofluorescence was from IEL. L, lumen of the artery. **n** Quantification of lesion area in LCA. **o** Quantification of plaque size determined with the Oil-Red-O-stained sections. **p**–**r** Quantification of VCAM1 (**p**), CD45 (**q**), and KLK10 (**r**) expression according to the immunostaining of the frozen sections. Data shown as mean ± s.e.m; *P < 0.05; **P < 0.01; ***P < 0.001; ns, *P* > 0.05 as determined by Student’s *t*-test

Following ultrasound treatment, the AAV gene vectors were injected and the transgene expression in ECs were determined with quantitative real-time PCR (qPCR) analysis (Fig. 1b, cand Supplementary Fig. S2). The ultrasound treatment significantly increases the transgene expression in LCA, which is positively related to the acoustic power and MB dose (Supplementary Fig. S3a). We chose the acoustic mechanical index (MI) at 0.4 and MB dose at 1 × 10^8^/mouse for further experiments. Under this condition, the transgene expression in LCA is over 24-fold higher than that in RCA on the 4th day post treatment. The immunostaining against EGFP also shows that EGFP was highly expressed in LCA ECs while low EGFP expression was found in RCA (Fig. 1d). We also determined the transgene expression in LCA ECs by varying the time interval between MBs and AAV injection (Supplementary Fig. S3b). We found that, when the time interval is in 3 h, the transgene expression in LCA was not significantly changed. In addition, no significant change in the expression of VCAM1, an inflammatory marker gene, was detected in LCA ECs, indicating the safety of UMGAAV (Supplementary Fig. S4). To further evaluate the persistence of transgene expression, the mice were sacrificed at different time points post UMGAAV. The qPCR analysis shows that transgene expression in LCA ECs increases from the 4th day and reaches the maximum in the 4th week (Fig. 1e). At the 8th week, the transgene expression in LCA ECs still remains a very high level. Interestingly, EGFP was also expressed in the medial and adventitial layer (M + A) of LCA. At the 4th week, the transgene expression in LCA M + A is ~86 times higher than that in RCA M + A, indicating that UMGAAV may also be used for gene delivery targeting other vascular cells such as the smooth muscle cells.

To better understand the underlying mechanism of UMGAAV, Evans Blue staining was applied. As expected, the ultrasound treatment increases the permeability of LCA, but the permeability can be recovered after 24 h (Supplementary Fig. S5). The degree of permeability change of the artery is related to the acoustic power of ultrasound. The area of ultrasound treatment can be controlled by adjusting the size of the color box in color Doppler imaging (Supplementary Fig. S6). The AAV gene vector was further labeled with fluorescent dyes to indicate the distribution of AAV in the artery (Fig. 1f and Supplementary Fig. S7). At 1 h, the signal of AAV in RCA was predominately detected in the EC layer in the form of big bright spots, while the signal in LCA was uniformly dispersed in both endothelium and medial layer. At 3 h, the signal in LCA remains very high, while no significant AAV signal could be detected in RCA. The signal in LCA is about 37 times higher than that in RCA at 3 h. These results explained why the transgene expression by the AAV gene vector is highly enhanced by ultrasound treatment. Without ultrasound treatment, most AAVs are initially bound to ECs as big aggregates. But this kind of binding is not stable and most of the bound AAV are flushed away by blood flow. When ultrasound treatment is applied, the permeability of the artery is increased. AAV can be well dispersed in the ECs and medial layer and more AAV will be internalized by cells.

In theory, UMGAAV can be used for the targeted gene delivery to all the arteries that can be visualized by ultrasound imaging. We performed UMGAAV in abdominal aorta and femoral artery. Under color Doppler ultrasound imaging, the suprarenal abdominal aorta (SAA) and infrarenal abdominal aorta (IAA) can be clearly visualized (Fig. 1g). As expected, ultrasound treatment can increase the permeability of the abdominal aorta (Supplementary Fig. S8). After MB injection, selective transgene expression in either of these two parts can be achieved (Fig. 1h). With a similar method, we can also selectively treat the major branches of the common femoral artery and robust transgene expression in the right femoral artery (RFA) was induced when RFA was treated alone (Fig. 1i, j and Supplementary Fig. S9).

The development of atherosclerosis is related to inflammation of artery ECs exposed to disturbed flow (d-flow). Kallikrein-10 (KLK10) is one of the atheroprotective genes that are downregulated by d-flow.^3^ We determined the feasibility of targeted delivery of KLK10 gene with UGMAAV in the mouse partial carotid ligation (PCL) model.^4^ Under the optimized condition (Supplementary Fig. S10), the KLK10 expression in LCA can be elevated to a level comparable to that in RCA (Fig. 1k and Supplementary Fig. S11) and the expression of VCAM1 was inhibited (Fig. 1l). We further tested whether UMGAAV-mediated KLK10 expression can reduce atherosclerotic lesions in the PCL model using ApoE^−/−^mice. With UMGAAV-mediated KLK10 delivery, the atherosclerotic lesion area development and immune cell filtration were significantly reduced (Fig. 1m). Compared with the saline control group, the atherosclerotic lesion area in LCA was decreased from 59.3 ± 12.5% to 28.9 ± 14.2% (Fig. 1n), the plaque size was decreased by 86.0% (Fig. 1o), the CD 45 expression was decreased by 77.9% (Fig. 1p), and the VCAM1 expression was decreased by 54.5% (Fig.1q), while the KLK10 expression was increased by 85.3% (Fig. 1r). Furthermore, lipid profiling showed no significant difference between the three groups (Supplementary Fig. S12).

Taken together, our results established proof of concept for UMGAAV as a paradigm for spatial control of transgene expression in mouse arteries. Compared with the method that delivers viral gene vectors into arterial endothelium via surgery,^2^ UMGAAV enables targeted noninvasive transgene expression and is applicable to several arteries that are inaccessible to surgery. In addition, the ultrasound imaging machine used for UMGAAV is a very common one that has been used in clinics. MBs have been used for contrast-enhanced ultrasound imaging in clinics. Considering that the AAV gene vectors have been investigated in clinical trials with promising results,^5^ UMGAAV could be clinically relevant.

## Supplementary information

Supporting information

## Data Availability

All relevant data are available in Supplementary Information and from the authors.
