# Supplementary material for: Spatial control of robust transgene expression in mouse artery endothelium under ultrasound guidance
Source: Signal Transduct Target Ther. 2022 Jul 18;7:225. doi: 10.1038/s41392-022-01031-w (PMC9288995; doi:10.1038/s41392-022-01031-w)
Supplement: Supplementary file 1 — Supporting information [file 41392_2022_1031_MOESM1_ESM.docx]

Supplementary Materials for

**Spatial control of robust transgene expression in mouse artery endothelium under ultrasound guidance**

Renfa Liu, ^#^ Shuai Qu, ^#^ Yunxue Xu, Hanjoong Jo, Zhifei Dai^*^

*Corresponding author. Email: [zhifei.dai@pku.edu.cn](mailto:zhifei.dai@pku.edu.cn)

**This PDF file includes:**

Supplementary Figs. S1 to S12

Supplementary Table S1

Materials and Methods


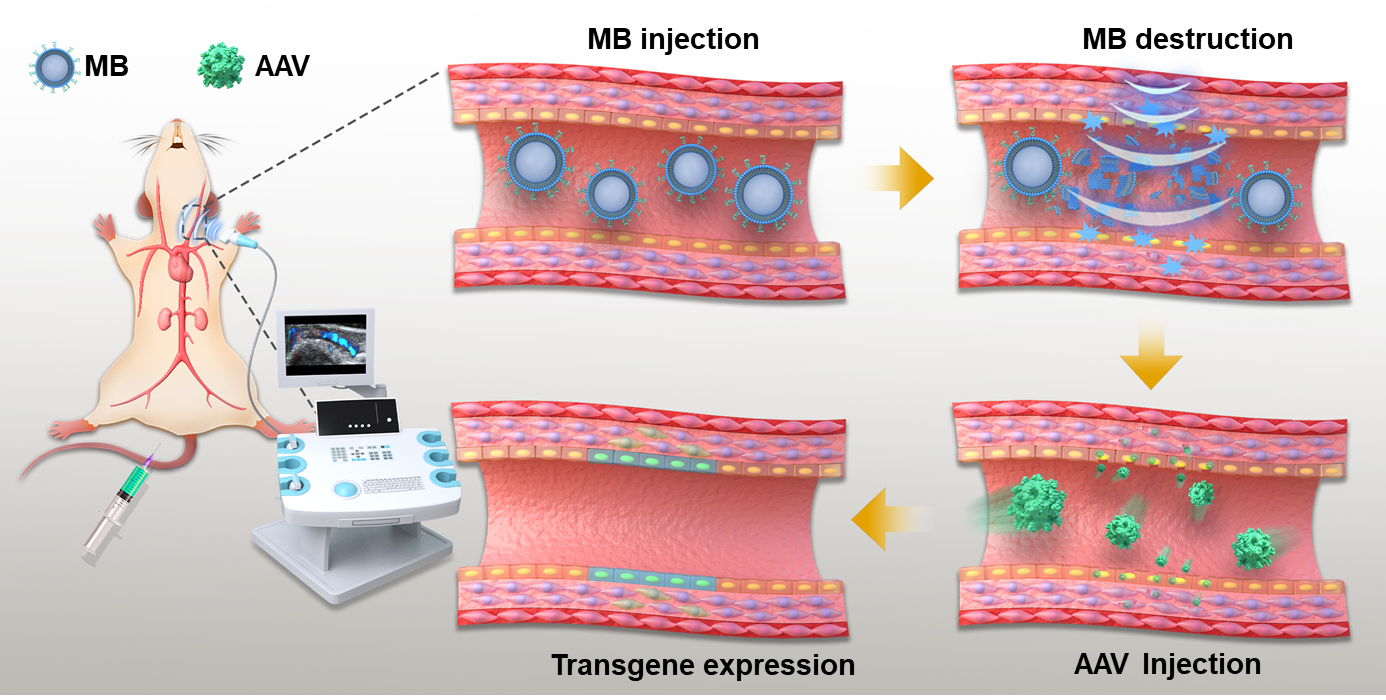


Supplementary Fig. S1.

**UMGAAV paradigm.** In the UMGAAV sequence, the mouse artery (e.g., carotid artery) can be visualized by ultrasound imaging. After injecting MBs, MBs can be selectively destroyed with ultrasound wave generated by the imaging probe under color Doppler mode, increasing the permeability of artery endothelium. Then the injected AAV can specifically accumulate in the ultrasound-treated area, resulting in targeted transgene expression in mouse artery.


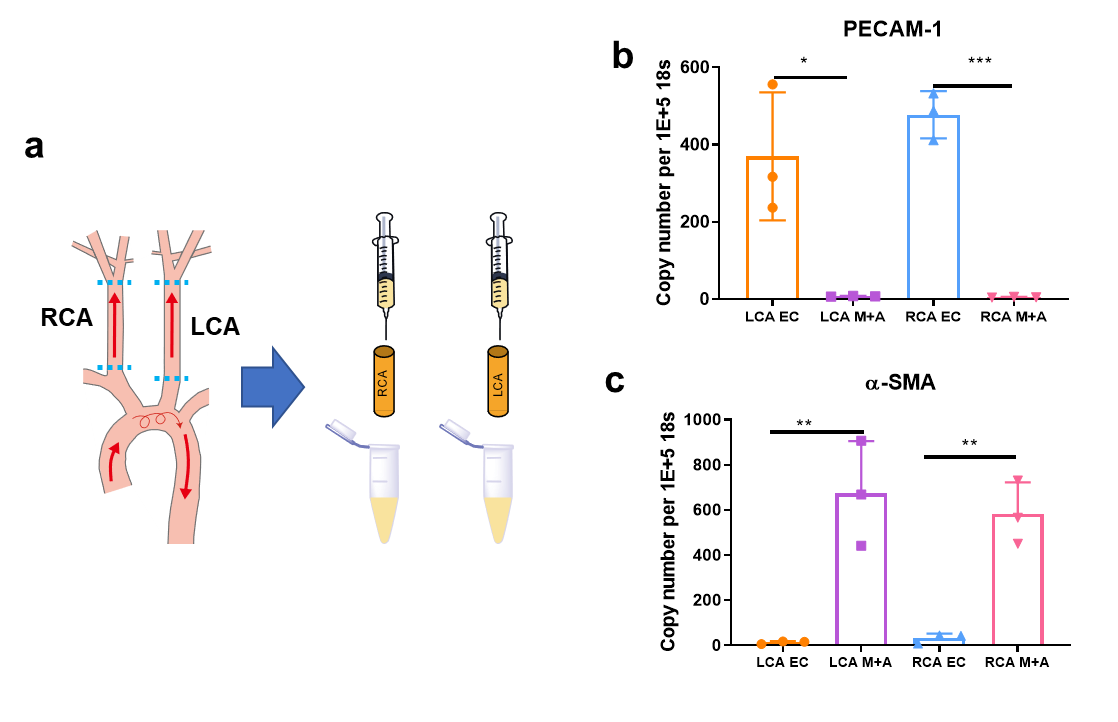


Supplementary Fig. S2.

**Isolating endothelial-enriched total RNA from mouse carotid arteries. a** Endothelial-enriched total RNAs were obtained by flushing the LCA and RCA. Following endothelial-enriched RNA preparation, the left-over samples representing media + adventitial (M+A) regions were also prepared. **b&c** To determine the purity of endothelial RNAs, qPCR was carried out using PECAM1 and αSMA, marker genes for endothelial and smooth muscle cells, respectively, and fold-change was determined by normalizing against 18S as an internal control. Data shown as mean±s.e.m; *P < 0.05; **P<0.01; ***P<0.001; ns, P>0.05 as determined by Student's t-test.


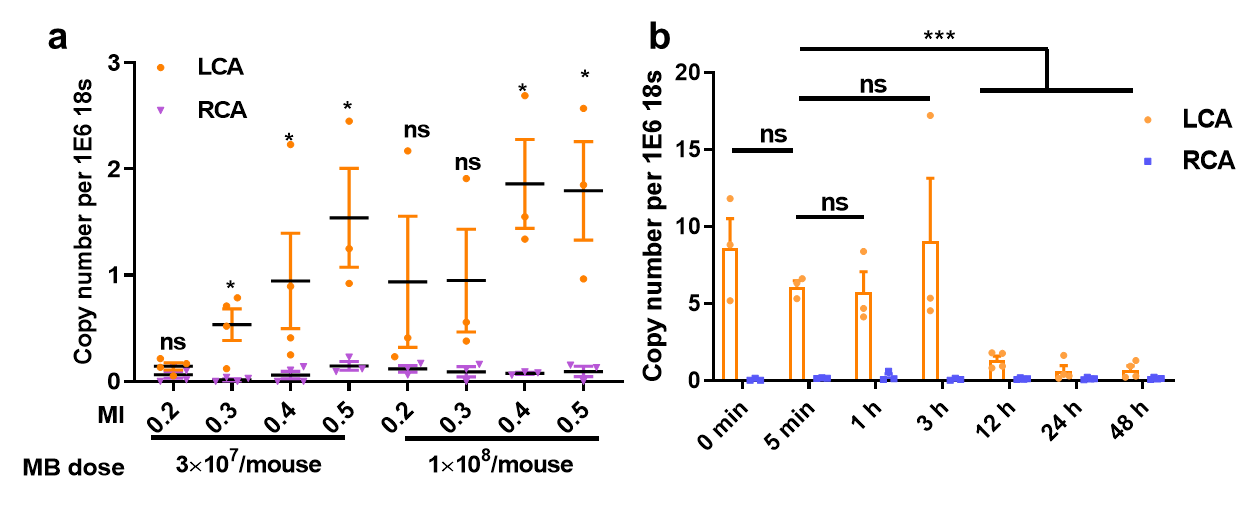


Supplementary Fig. S3.

Optimizing parameters in UMGAAV procedure. **a** Transgene expression of EGFP in endothelium of LCA and RCA by varying ultrasound mechanical index (MI) and MB dose. The time interval between MB and AAV injection was ~5 min. The mice were sacrificed at the 4^th^ day post AAV injection. **b** Transgene expression of EGFP in endothelium of LCA and RCA by varying time interval between MB and AAV injection. The mice were sacrificed at the 4^th^ day post AAV injection. Data shown as mean±s.e.m; *P < 0.05; **P<0.01; ***P<0.001; ns, P>0.05 as determined by Student's t-test.

Supplementary Fig. S4.

VCMA1 expression in carotid endothelium. After injecting MBs at different dose, the LCA was treated with ultrasound at different mechanical index (MI) for 30s. The time interval between MB and AAV injection was ~5 min. The mice were sacrificed at the 4^th^ day post AAV injection. The endothelial-enriched RNA samples were then used to determine VCAM1 expression with qPCR and fold-change was determined by normalizing against 18S as an internal control. Data shown as mean±s.e.m; ns, P>0.05 as determined by Student's t-test.


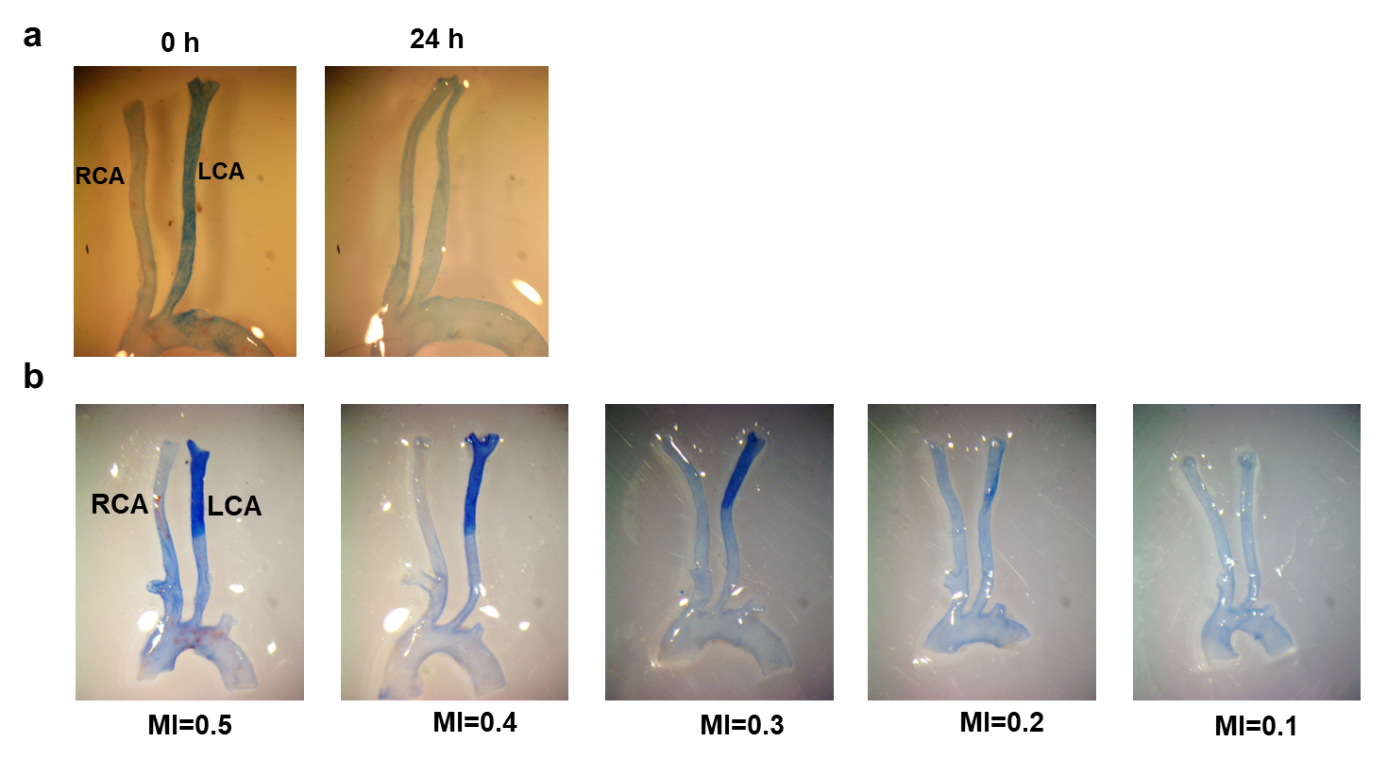


Supplementary Fig. S5.

Ultrasound triggers transgene expression by increasing arterial permeability. **a** Representative bright-field images of artery trees from mice treated with ultrasound in LCA. Evans Blue was injected intravenously immediately or 24 h post ultrasound treatment. The mice were sacrificed 30 min post Evans Blue injection and artery trees were dissected out for bright field imaging. **b** Permeability changes of mouse carotid artery as function of acoustic power. After MBs injection, ultrasound treatment with different MI was applied to the top part of LCA. 100 μL Evans Blue (1% in saline) was then injected intravenously. The mice were sacrificed 30 min later and the isolated artery trees were then observed under bright field microscope. The degree of blue color in the treated area directly reveals the permeability changes by ultrasound treatment.


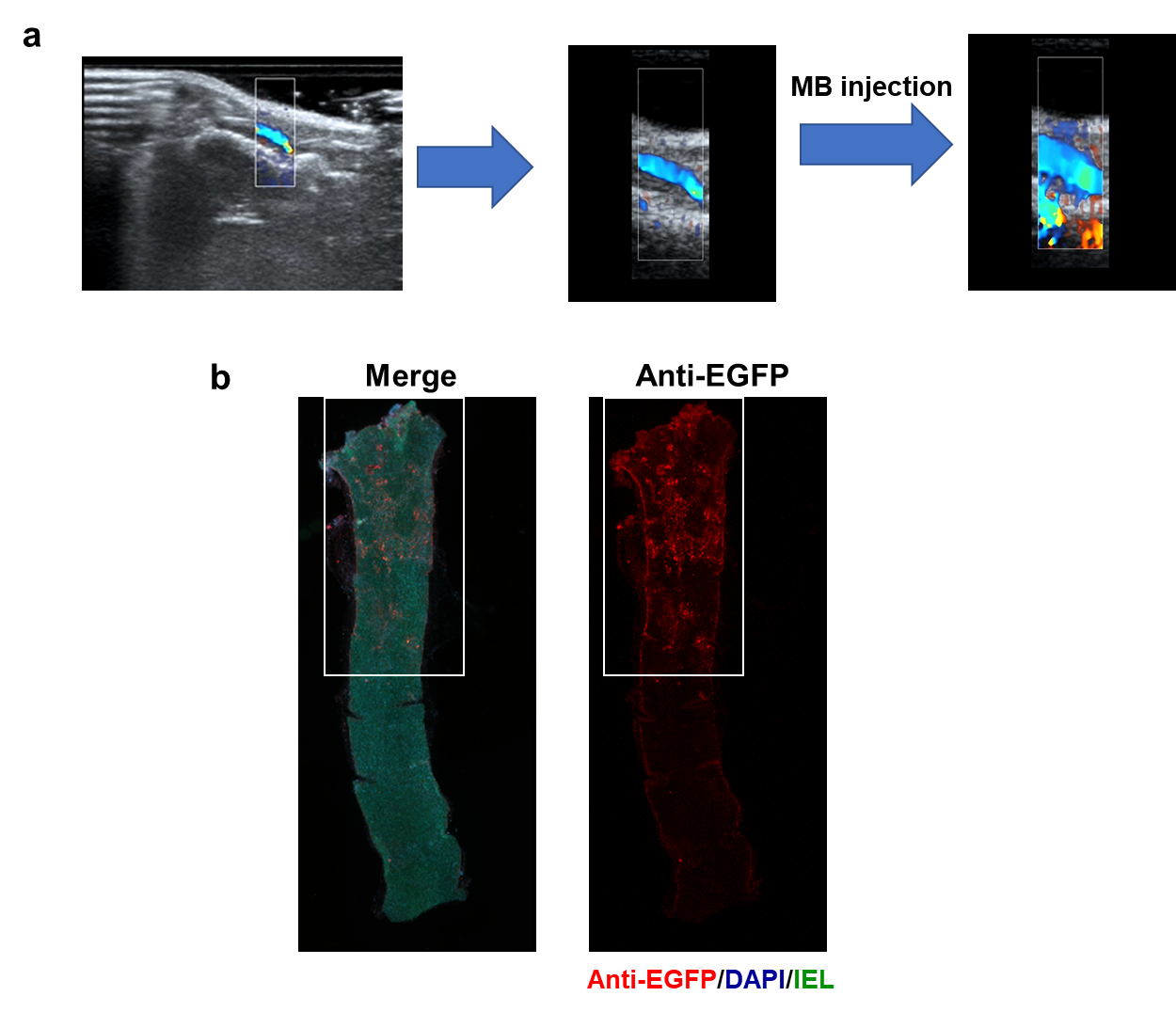


Supplementary Fig. S6.

Spatial control of transgene expression in mouse carotid artery. **a** The mouse carotid artery can be visualized with ultrasound imaging under color Doppler mode. The position and size of color box can be adjusted to the top part of the carotid artery (the left picture). After zooming the image to the selected area, the ultrasound will be focused to this area (the middle picture). Following MBs injection, the MBs can be destroyed as evidenced by the color “blooming” phenomenon (the right picture). **b** After ultrasound treatment, AAV gene vector encoding EGFP was injected. The mouse was sacrificed at the 4^th^ day and the carotid artery was *en face* stained with anti-EGFP antibody (red). The DAPI staining marks cell nuclei (blue). The green autofluorescence was mainly from the internal elastic lamina (IEL). The EGFP expression was primarily detected in the top half part of the carotid artery.


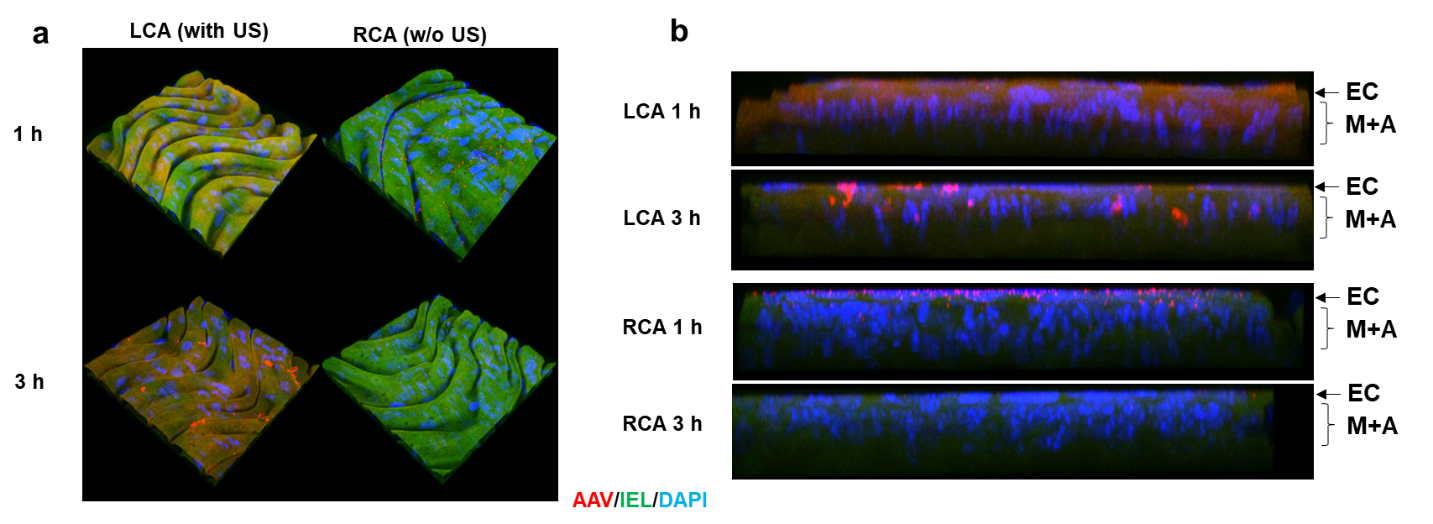


Supplementary Fig. S7.

Representative confocal images of carotid artery from two different angles of 3-D views. After treating LCA with ultrasound and injecting AAV labeled with red fluorescence, the mice were sacrificed at 1 h and 3 h post AAV injection. The DAPI staining marks cell nuclei (blue). The green autofluorescence was from the IEL.


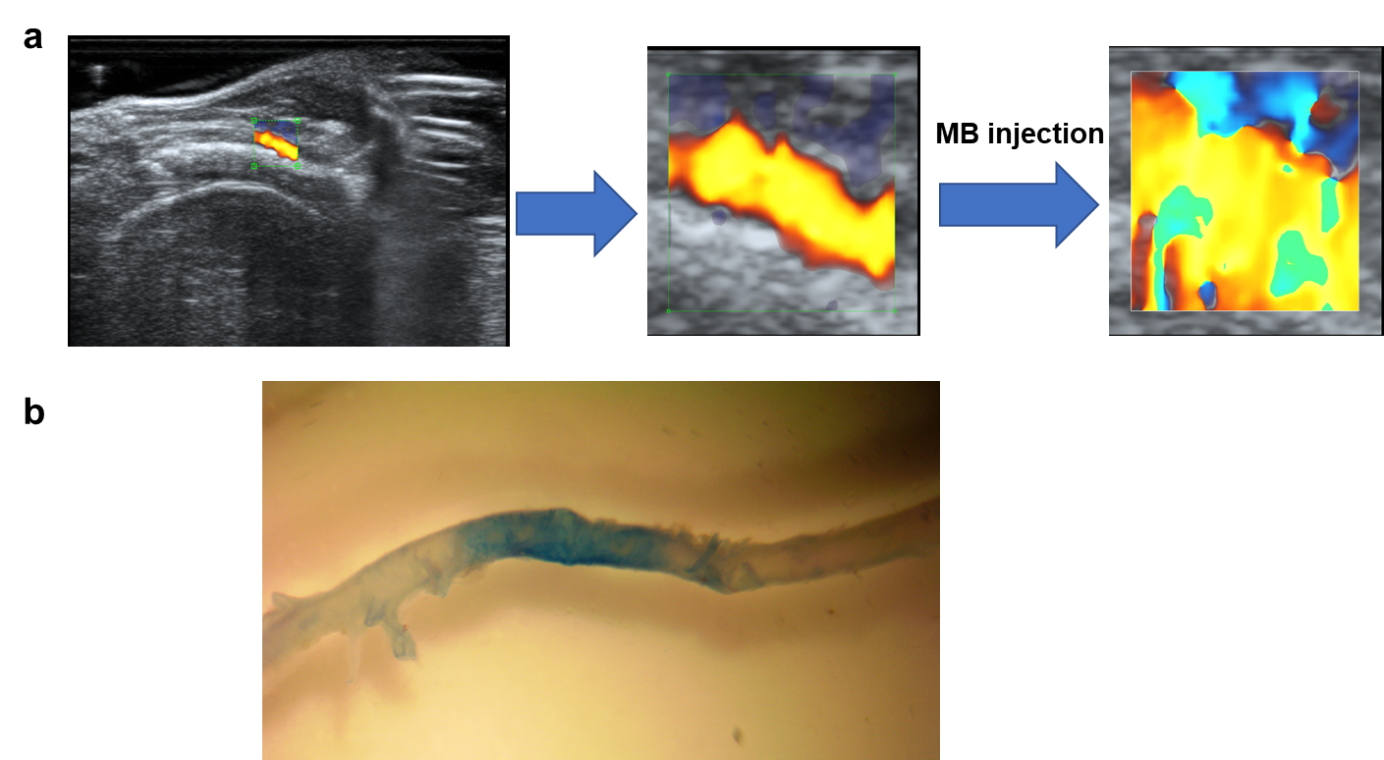


Supplementary Fig. S8.

Ultrasound-mediated permeability changes in mouse abdominal aorta. **a** The mouse abdominal aorta can be visualized with ultrasound imaging under color Doppler mode. The position and size of color box can be adjusted to a very small area (the left picture). After zooming the image to the selected area, the ultrasound will be focused to this area (the middle picture). Following MBs injection, the MBs can be destroyed as evidenced by the color “blooming” phenomenon (the right picture). **b** After the ultrasound treatment, 100 μL Evans Blue (1% in saline) was injected intravenously. The mice were sacrificed 30 min later and the abdominal aorta were then observed with bright field microscope. The degree of blue color in the treated area reveals the permeability changes by ultrasound treatment.


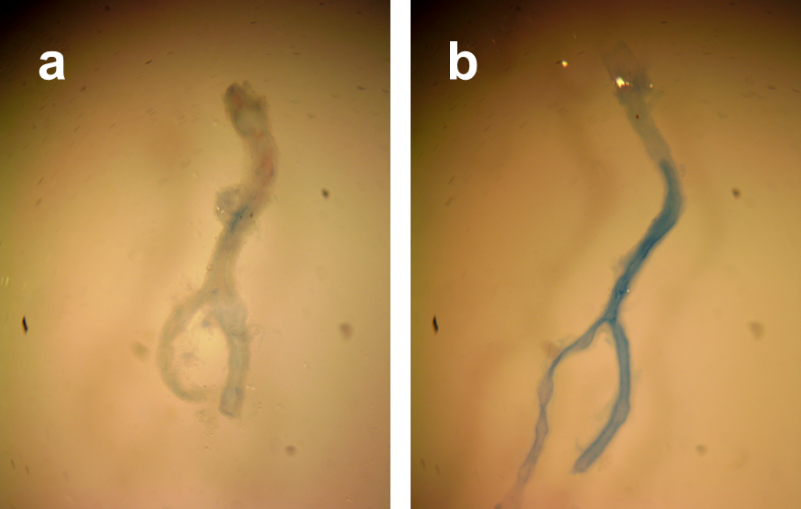


Supplementary Fig. S9.

Evans Blue staining of mouse femoral artery. After MB injection, ultrasound treatment was applied to the right femoral artery (RFA), while the left femoral artery (LFA) was not treated. 30 min post Evans Blue injection, the LFA (**a**) and RFA (**b**) was dissected and observed under the bright field microscope. The degree of blue color in the treated area reveals the permeability changes by ultrasound treatment.


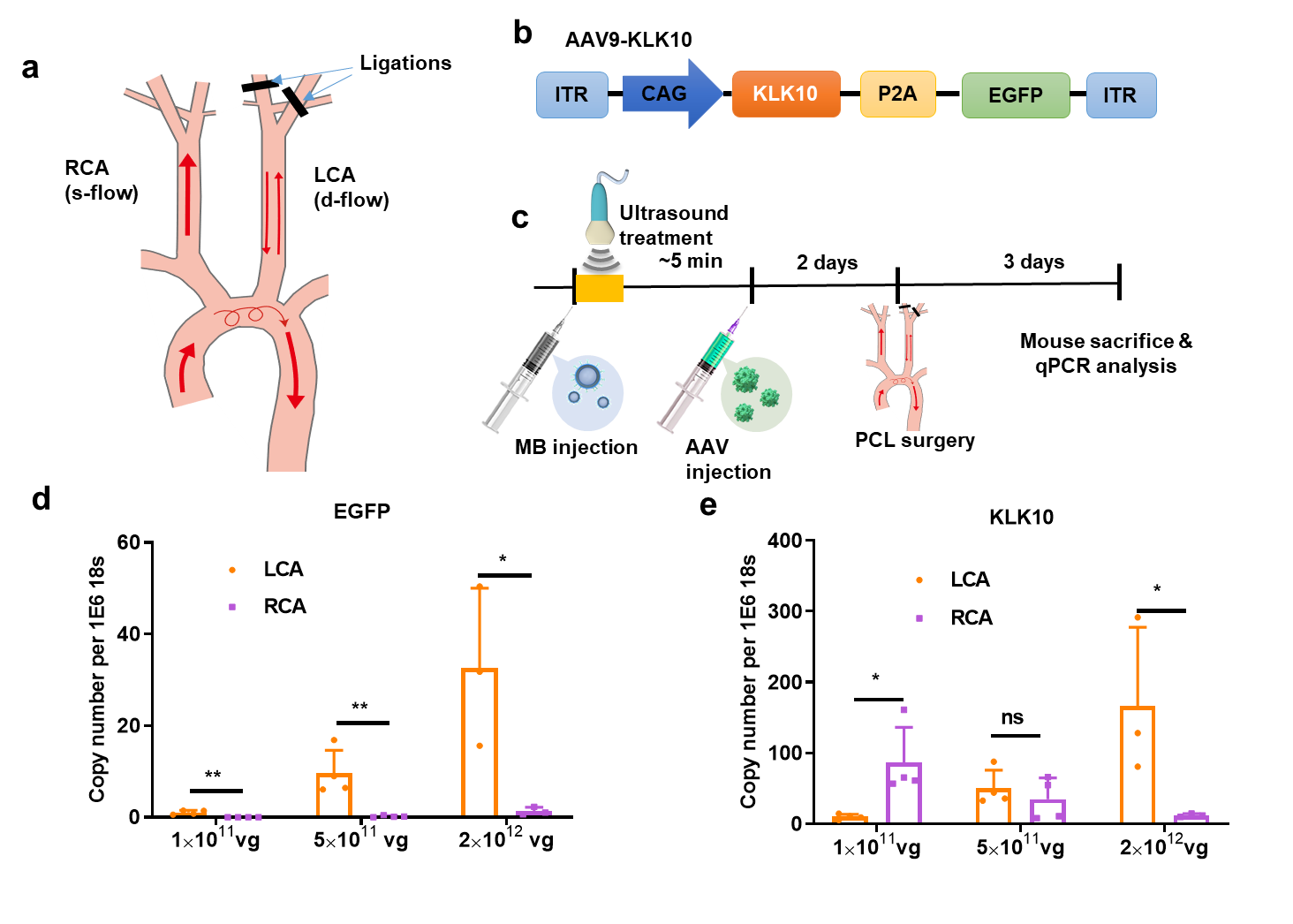


Supplementary Fig. S10.

UMGAAV for the targeted delivery of KLK10. **a** Schematic illustration of the surgically induced d-flow in partial carotid ligation model. Three of the four branches of LCA were ligated, while the contralateral RCA remains untouched as an internal control. **b** Structure of AAV9 gene vector expressing KLK10. ITR, internal terminal repeats; CAG, a hybrid construct consisting of the cytomegalovirus (CMV) enhancer fused to the chicken beta-actin promoter; P2A (porcine teschovirus-1 2A) peptide, a “self-cleaving” peptide, which undergoes self-cleavage to generate mature viral proteins by a translational effect that is known as “stop-go” or “stop-carry”. **c** Paradigm of protocols to determine effect of UMGAAV-mediated KLK10 delivery in PCL model. After MB injection, ultrasound irradiation was applied to LCA for 30 seconds. Two days following AAV injection, PCL surgery was conducted. The mice were sacrificed for qPCR analysis at the 3^rd^ day post-surgery. **d** Transgene expression of EGFP in endothelium of LCA and RCA at different AAV dose. **e** Expression of KLK10 in endothelium of LCA and RCA at different AAV dose. Based on these results, AAV dose at 5×10^11^ vg (vector genomes) per mouse was selected for further research. Data shown as mean±s.e.m; *P < 0.05; **P<0.01; ns, P>0.05 as determined by Student's t-test.


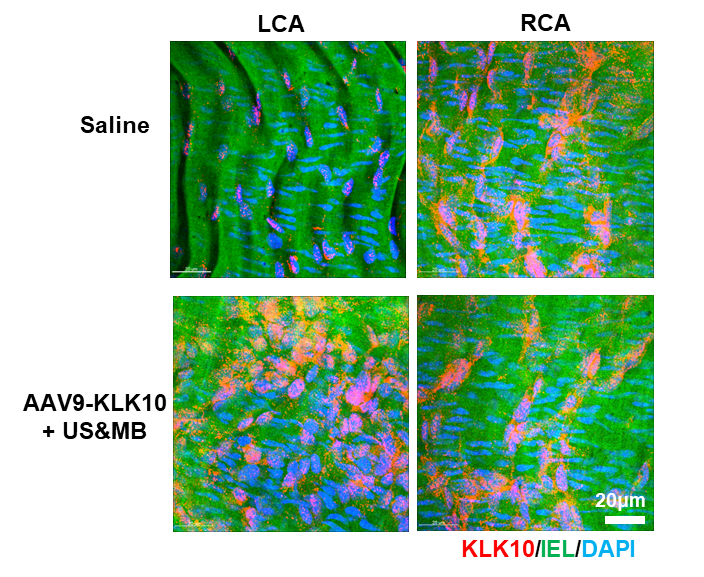


Supplementary Fig. S11.

*En face* KLK10 staining of carotid artery. The mice in the AAV9-KLK10+US&MB group were injected with AAV9-KLK10 after ultrasound treatment to LCA, while the mice in the saline group were injected with saline only. Two days after the treatments, PCL surgery was conducted. The mice were then sacrificed three days post-surgery. The carotid arteries were *en face* immunostained with KLK10 antibody (red). The DAPI staining marks cell nuclei (blue). The green autofluorescence was from IEL.


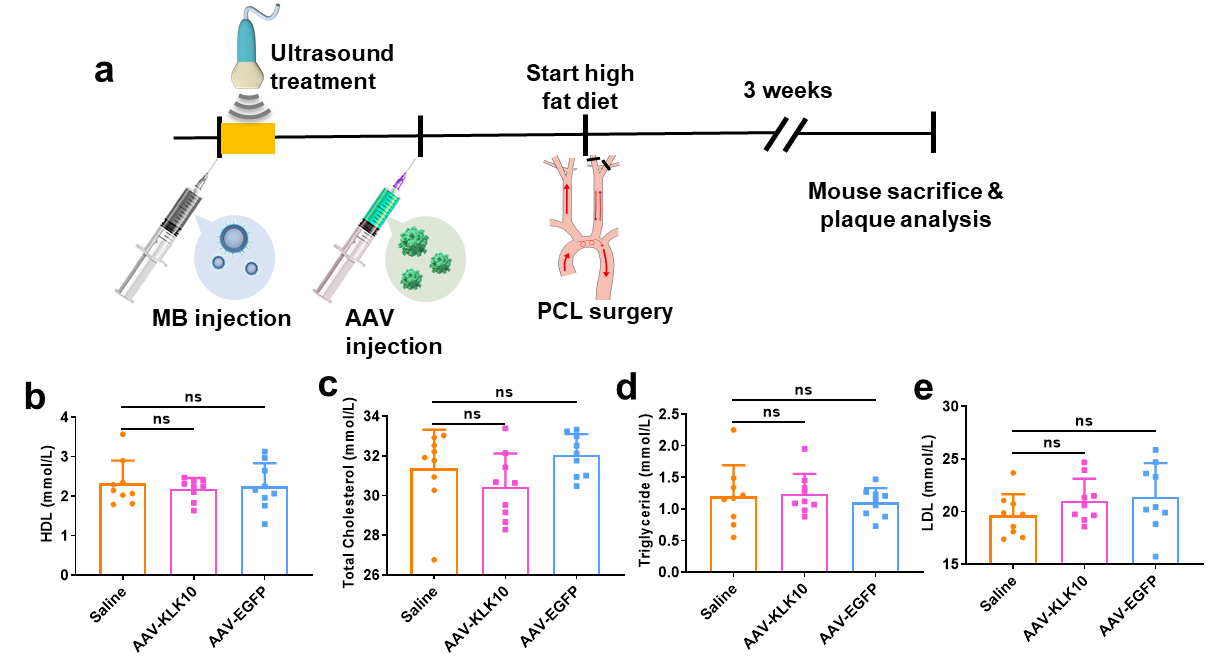


Supplementary Fig. S12.

UMGAAV-mediated KLK10 expression inhibit atherosclerosis. **a** Illustration of UMGAAV-mediated anti-atherosclerosis protocol. After MB injection, ultrasound irradiation was applied to LCA for 30 seconds. Two days following AAV injection, the mice were subject to PCL surgery. After fed with high fat diet for three weeks, the mice were sacrificed for plaque analysis. The mice were divided into three groups: Saline, mice were injected with saline only; AAV9-EGFP, the mice were injected with AAV9 expressing EGFP after ultrasound treatment; AAV9-KLK10, the mice were injected with AAV9-KLK10 after ultrasound treatment. **b-e** Serum lipid profiles. Blood was collected immediately following sacrifice and the plasma samples were then assayed to measure high-density lipoprotein (HDL), total cholesterol, triglycerides, and low-density lipoprotein (LDL). Data shown as mean±s.e.m; *P < 0.05; **P<0.01; ***P<0.001; ns, P>0.05 as determined by one-way ANOVA.

Supplementary Table S1.

**Primers for qPCR analysis**

| 18s(F) | CGCCGCTAGAGGTGAAATTCT |
| --- | --- |
| 18s(R) | CGAACCTCCGACTTTCGTTCT |
| PECAM-1(F) | ACGCTGGTGCTCTATGCAAG |
| PECAM-1(R) | TCAGTTGCTGCCCATTCATCA |
| α-SMA(F) | TTTCCAAATCATTCCTGCCC |
| α-SMA(R) | CGCTCTCAAATACCCCGTTT |
| EGFP(F) | AAGCTGACCCTGAAGTTCATCTGC |
| EGFP(R) | CTTGTAGTTGCCGTCGTCCTTGAA |
| VCAM-1(F) | TCTTGGGAGCCTCAACGGTA |
| VCAM-1 (R) | CAAGTGAGGGCCATGGAGTC |
| KLK10(F) | CGCTACTGATGGTGCAACTCT |
| KLK10(R) | CAGTGGCTTATTTCTCCAGCAA |

**Materials and Methods**

**Microbubble preparation**

The microbubbles are prepared with a modified mechanical agitation method established in our lab. The method is described as following: Dissolve DSPC and DSPE-mPEG2000 (Shanghai A.V.T. Pharmaceutical) in ethanol at the concentration of 10 mM. Add 1800 μL DSPC and 200 μL DSPE-mPEG2000 solution into a 20 mL glass vial and mix. The ethanol is evaporated under nitrogen gas flow at 80 °C. 1 mL propylene glycol (Sigma–Aldrich) and 1.26 g glycerol (Sigma–Aldrich) are then to fully dissolve the lipids at 60 °C. Under vigorous stirring, 8 mL PBS (pre-warmed at 60 °C) is added to the lipid solution. After cooled to room temperature, the solution is sterilized with 0.22 μm syringe filter and sub-packed in 2-mL glass vials (1 ml/vial). The headspace of the vial is filled with perfluoropropane gas. The solution can be stably stored at 4 °C for several months. Before use, the vial is shaken with the Vialmix (Lantheus) for 45 s to get the microbubbles. The concentration of microbubbles can be quantified with cytometry. The concentration of microbubbles is around 1.2×10^10^/mL.

**AAV packaging**

Recombinant AAV serotype-9 expressing EGFP under the control of CAG promoter, a hybrid construct consisting of the cytomegalovirus (CMV) enhancer fused to the chicken beta-actin promoter are produced by Vigene Biosciences (Jinan, China). Plasmid encoding KLK10 is custom-constructed by GENEWIZ (Suzhou, China) and then packed into AAV9 by Vigene Biosciences.

**Animals**

C57BL/6 mice and ApoE^-/-^ mice were obtained from Charles River Labs (Beijing, China). Animals were housed in a 12 h light/dark cycle and were provided with water and food ad libitum. All experiments were conducted under a protocol approved by the Institutional Animal Care and Use Committee of the Peking University.

**General protocol of UMGAAV**

All ultrasound imaging treatments were conducted using a DC-8 ultrasound system equipped with a L12-3E linear-array probe (Mindray). Mice were anesthetized with inhaled isoflurane, and body temperature was maintained on a heated stage for the duration of studies. The position of left carotid artery or abdominal aorta can be found under B-mode ultrasound imaging. Then switch to color Doppler mode. Change the size and position of color box to the region of interest, zoom to the selected area and then change mechanical index (MI) to desired value. Immediately after microbubbles injection, press the “Freeze” button every second so that ultrasound is applied in an ON/OFF mode (1 s ON followed by 1 s OFF) for a total time of 30s.

For the UMGAAV of femoral artery, the ultrasound probe was placed in parallel with the femoral artery. Switch the ultrasound imaging to the contrast mode. Inject microbubbles and find the position of femoral artery under the contrast mode. Change the size and position of box to the region of interest and zoom to the selected area. Switch the ultrasound imaging to the color Doppler mode and treat the selected area with a MI of 0.4. Press the “Freeze” button every second so that ultrasound is applied in an ON/OFF mode (1 s ON followed by 1 s OFF) for a total time of 30s.

After the ultrasound treatment, the AAV encoding gene of interest will be injected intravenously through the penile vein. At certain time point post the treatment, the mice are sacrificed for further characterization of gene expression.

**Isolation of Endothelial-Enriched RNA**

Endothelial-enriched RNA from the carotid arteries was extracted as previously described^1^. Briefly, mice were killed by CO_2_ inhalation and perfused with saline via the left ventricle after severing the inferior vena cava. The LCA and RCA were then isolated and carefully cleaned. The carotid lumen was quickly flushed with 100 μl QIAzol lysis reagent (QIAGEN) using a 29-gauge insulin syringe in a microfuge tube. The eluate was then used for intimal RNA isolation using an RNA MiniPrep kit (ZYMO research) according to the manufacturer’s instructions. The carotid artery leftover after flushing with QIAzol was used to prepare RNA from M+A. M+A was placed in QIAzol lysis reagent (350 μl per carotid), and homogenized with a homogenizer. Marker genes (PECAM-1 and α-SMA) was used to determine the purity of endothelial RNA in each prep (Supplementary Fig. S1).

**qPCR**

RNA samples were reverse-transcribed with the PrimeScript RT reagent kit (TAKARA) and qPCR was performed on selected genes using the SYBR Premix Ex Taq (TAKARA) with custom-designed primers using 18S as house-keeping control. The Bio-rad CFX96 Touch qPCR machine was used for qPCR measurements. The PCR conditions were 30 s at 95 °C, followed by 40 cycles of 95 °C for 5 s and 60 °C for 30 s. Fold changes between LCA and RCA were determined for all targets using the ΔΔCt method. Sequences for primers used in this study were listed in Supplementary Table S1.

***En face* staining**

The carotid arteries are excised, fixed in 4% paraformaldehyde, penetrated with 0.025% Triton X-100 for 10 min, blocked with 10% goat serum for 2 h at room temperature, (RT) and incubated with primary anti-EGFP (abcam ab6556), anti-VCAM1 (abcam, ab134047) or anti-KLK10 (Boster, BA3803-2) antibodies overnight at 4 °C. To visualize primary antibodies, Alexa Fluor ® 594-conjugated secondary antibody (abcam ab150080) was used for 1 h at RT. Nuclei were counterstained with DAPI. Samples were imaged using a Nikon confocal microscope.

**Evans Blue staining**

The permeability changes of the artery can be determined by Evans Blue. At pre-determined time points post ultrasound treatment, inject 100 μL Evans Blue (1% in saline) intravenously and sacrifice the mouse 30 min later. The carotid arteries are then dissected and observed under microscope. The degree of blue color in the treated area directly reveals the permeability changes by ultrasound treatment.

**Fluorescence tracking of AAV**

The AAV was modified with fluorescent dyes following a procedure reported in the literature with some modification.^2^ In brief, purified AAV9 was incubated for 1 hour at 4 °C in PBS with tenfold excess of Sulfo-Cyanine3 NHS ester over the capsid protein units (each AAV is composed of 60 capsid protein units). Labelled viruses were separated from the free dyes by dialysis against PBS at 4 °C and stored at −80 °C in small aliquots. Following ultrasound treatment to LCA, 5×10^11^ vg/mouse labelled viruses was injected intravenously. At predetermined time points post injection, the mice were sacrificed. The carotid arteries were dissected, fixed with formalin, and stained with DAPI. Samples were then subjected to *en face* confocal imaging with a Nikon confocal microscope.

**Mouse partial carotid ligation surgery**

The partial carotid ligation surgery was conducted as previously reported.^1^ Mice at 8–9 weeks of age were anaesthetized with 3.5% isoflurane initially and then ~2% during the entire procedure. Before surgery, analgesic carprofen (5mg/kg) was administrated subcutaneously. The LCA bifurcation was exposed by blunt dissection and three caudal branches of LCA (left external carotid, internal carotid, and occipital arteries) were ligated with 6-0 silk sutures while leaving the superior thyroid artery intact. For atherosclerotic studies, the ApoE^-/-^ mice were fed with a Paigen’s high fat diet (15.8% fat, 1.25% cholesterol, 0.5% sodium cholate) following the surgery until killed. C57BL/6 mice were continued with a chow diet.

**Serum lipid analysis**

Serum lipid analysis was performed by the department of laboratory animal science in Peking University Health Science Center for total cholesterol, triglycerides, HDL, and LDL.

**Plaque lesion analysis**

Carotid arteries were isolated *en bloc* from ligated ApoE^-/-^ mice fed with a high fat diet for 3 weeks. The artery trees were photographed using a dissection microscope and the opaque area covered by plaque and total area of LCA were quantified using NIH ImageJ software.

**Immunohistochemistry**

Arteries were embedded in optimal cutting temperature compound (Tissue-Tek), frozen on dry ice and stored at -80 °C until used. To visualize atherosclerosis development, Oil-Red-O staining was carried out. Frozen sections were fixed in formalin for 10 min, rinsed with water and 60% propylene glycol, and then stained with Oil-Red-O staining solution (Solarbio). Slides were then differentiated in 60% propylene glycol, rinsed with distilled water, and counterstaining was done using Mayer’s haematoxylin (Beijing Leagene Biotechnology Co., Ltd.) for 5 min.

For VCAM1 and CD45 staining, sections were fixed in a 1:1 mixture of methanol/acetone for 10 min at RT and then blocked using 10% (v/v) goat serum (2 h, at RT). Immunohistochemical staining was conducted anti-VCAM1 (Abcam ab134047) or anti-CD45-biotin (eBioscience) overnight at 4 °C. Secondary staining for VCAM1 was performed using Alexa Fluor ® 594-conjugated goat anti-rabbit antibody (Abcam ab150080) while Alexa Fluor ® 594-conjugated streptavidin (Bioss) was used for CD45. Nuclei were counterstained with DAPI. Samples were imaged using an Axio Scan.Z1 Slide Scanner (Zeiss).

**Statistical Analysis**

GraphPad Prism (GraphPad Software) was used for all the statistical analysis. Pairwise comparisons were performed using one-way Student’s t-tests. Multiple comparisons of means were performed using one-way analysis of variance (ANOVA) followed by Tukey's multiple comparison tests. All data with P<0.05 was considered statistically significant (*P<0.05; **P<0.01; ***P<0.001; ns, not significant (P>0.05)).

**References**

1 Nam, D. *et al.* Partial carotid ligation is a model of acutely induced disturbed flow, leading to rapid endothelial dysfunction and atherosclerosis. *Am. J. Physiol. Heart Circ. Physiol.* **297**, H1535-H1543 (2009).

2 Xiao, P. J., Li, C. W., Neumann, A. & Samulski, R. J. Quantitative 3D Tracing of Gene-delivery Viral Vectors in Human Cells and Animal Tissues. *Mol. Ther.* **20**, 317-328 (2012).
